# Clinical and MRI Restoration of ACL Continuity Following Functional Complete ACL Rupture in an Adolescent Athlete: A Case Report

**DOI:** 10.1002/ccr3.73465

**Published:** 2026-09-03

**Authors:** Hooman Angoorani, Molood Jafari Fesharaki, Chakavak Valadkhani, Hossein Safaei

**Affiliations:** ^1^ Department of Sports and Exercise Medicine, School of Medicine Hazrat‐e Rasool General Hospital, Iran University of Medical Sciences Tehran Iran

**Keywords:** ACL healing, adolescent athlete, case report, knee injury, spontaneous ligament restoration

## Abstract

Restoration of ACL continuity and clinical stability may occur in selected adolescents with functional ACL insufficiency. This case highlights the potential value of repeat clinical and MRI assessment before proceeding with definitive ACL reconstruction when clinical circumstances permit.

## 1 Introduction

The anterior cruciate ligament plays a critical role in maintaining knee stability during pivoting and cutting activities. ACL rupture is one of the most common serious knee injuries in young athletes [1]. It is often associated with instability, inability to return to sport, and long‐term risk of meniscal injury and osteoarthritis [2, 3]. Surgical reconstruction is widely accepted as the standard of care for physically active adolescents and young adults [4, 5]. Nevertheless, emerging studies have challenged the traditional belief that the ACL lacks the capacity to heal, reporting cases of partial or complete ligament healing [6, 7].

While spontaneous ACL healing has been reported in select adult populations, including sedentary individuals managed conservatively [8, 9, 10], reports in adolescents remain rare and clinically controversial, and factors guiding safe nonoperative decision‐making in adolescents are not well defined [11].

Although a few case reports have documented intrinsic ACL healing in skeletally immature patients, including an adolescent reported by Bagsby et al. and an 8‐year‐old child reported by Arts et al., such evidence remains extremely limited [12, 13]. Besides potential biological differences in regenerative capacity, adolescents, particularly active individuals, face higher mechanical demands on the knee, including increased rotational and pivoting stresses, which may influence healing and clinical outcomes [14, 15].

**FIGURE 1:**
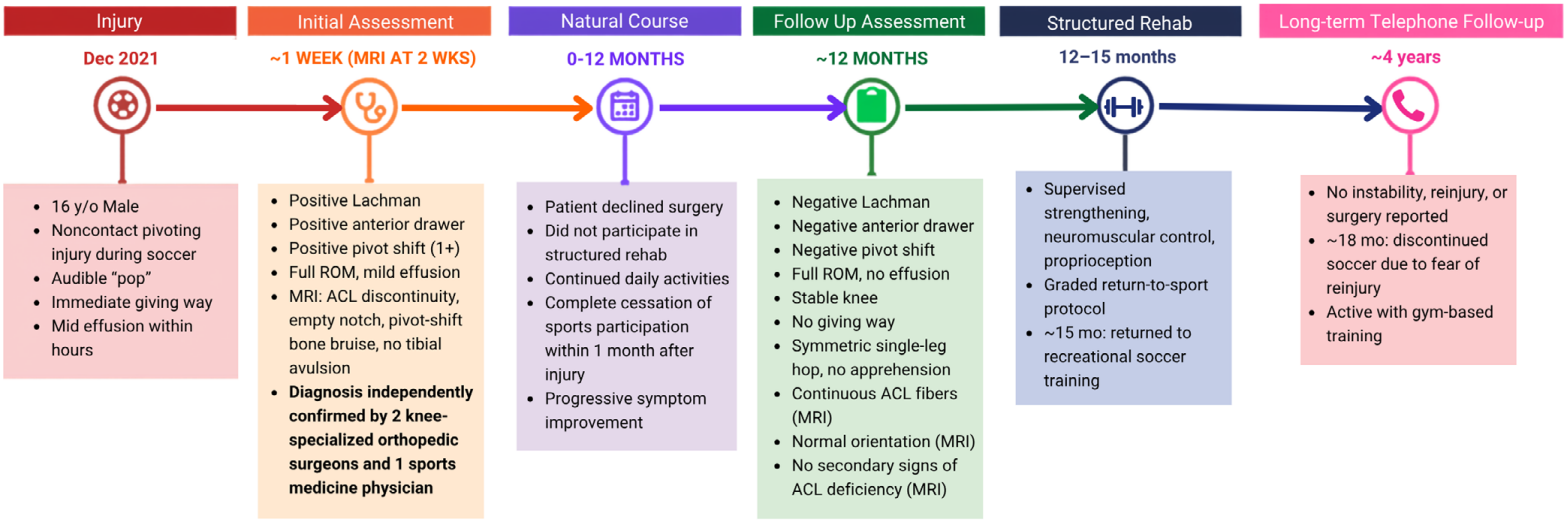
Chronological sequence of injury, assessment, management, and outcome. A 16‐year‐old soccer player sustained a noncontact pivoting injury with clinical and MRI evidence of functional complete ACL rupture. After declining reconstruction, he improved without surgery. At ~12 months, clinical examination was stable and MRI showed restored ACL continuity. Structured rehabilitation and graded return to sport followed, with return to soccer training at ~15 months. Telephone follow‐up at ~4 years revealed no reported instability, reinjury, or surgery; he had discontinued soccer because of fear of reinjury and remained active with gym‐based training.

The chronological sequence of clinical events, imaging evaluations, management decisions, and follow‐up assessments is summarized in Table 1 and Figure 1.

**TABLE 1:** Timeline of clinical events, diagnostic evaluations, and follow‐up in an adolescent athlete with functional anterior cruciate ligament rupture and subsequent clinical and radiologic recovery.

Time	Clinical course/event
December 2021	Non‐contact pivoting injury during recreational soccer
1 week	Initial sports medicine evaluation: positive Lachman, anterior drawer, and grade 1 pivot‐shift; functional ACL insufficiency diagnosed
January 2022 (~2 weeks)	MRI demonstrated loss of ACL fiber continuity and findings suggestive of complete ACL rupture; ACL reconstruction recommended
0–12 months	Patient declined surgery, did not participate in structured rehabilitation, progressively reduced sports participation, and ultimately stopped playing soccer
12 months	Re‐evaluation demonstrated resolution of clinical instability (negative Lachman, anterior drawer, and pivot‐shift); repeat MRI showed restoration of ACL continuity; reconstruction deferred
12–14 months	Supervised rehabilitation focusing on strength, neuromuscular control, proprioception, and progressive return‐to‐sport training
15 months	Completed a graded return‐to‐sport program and returned to non‐competitive soccer training without pain, subjective instability, or recurrent episodes of giving way; ACL reconstruction remained unnecessary
~4 years	Structured telephone follow‐up: patient continued soccer training for approximately 3 months following his 15‐month return, then voluntarily discontinued soccer at approximately 18 months post‐injury because of fear of reinjury rather than knee symptoms or instability; thereafter remained physically active with gym‐based resistance training and general fitness; no recurrent knee instability, swelling, reinjury, or ACL reconstruction

## 2 Case Presentation

### 2.1 Patient Information and History of Present Illness

A 16‐year‐old male recreational soccer player presented to the Iran Football Medical Assessment and Rehabilitation Centre (IFMARC), Tehran, Iran, 1 week after sustaining a right knee injury during a soccer activity. The injury occurred in December 2021.

The injury occurred during a noncontact pivoting maneuver while running backward with the right foot planted, involving a rapid change of direction and internal rotation of the tibia relative to the femur. During this movement, the patient heard and felt a distinct audible “pop” in the knee. Immediately after the incident, he experienced a subjective sensation of the knee giving way but was able to continue playing cautiously despite discomfort. Mild knee effusion developed within several hours after the injury, without substantial swelling.

He had no prior history of knee injury, ligamentous instability, or lower extremity surgery. His past medical history was unremarkable, and he was not taking any regular medications. There was no family history of connective tissue disorders or joint instability. He was a recreational soccer player, training approximately two to three times per week.

During the first several hours after injury, he did not notice significant knee swelling or stiffness and did not seek emergency department care. He did not use a brace, crutches, or immobilization device and managed symptoms with relative rest only, without regular use of nonsteroidal anti‐inflammatory medications. Over the following week, he attempted to continue soccer on an intermittent basis but reported episodes of instability and pain, particularly during cutting, pivoting, and rapid deceleration.

### 2.2 Physical Examination

On physical examination 1 week after the injury, no erythema, ecchymosis, deformity, or gross malalignment was noted. There was no quadriceps atrophy compared with the contralateral side.

A mild joint effusion was appreciated on sweep testing, whereas the patellar tap test was negative. Palpation revealed no focal tenderness along the medial or lateral joint lines, femoral condyles, tibial plateau, or patellar facets.

The active and passive range of motion of the right knee was full and symmetric compared with the contralateral side, with extension to 0° and flexion beyond 135°. Mild pain was reported at terminal flexion.

Ligamentous examination demonstrated a positive Lachman test, characterized by increased anterior tibial translation with a soft endpoint compared with the contralateral knee. The anterior drawer test was likewise positive, demonstrating increased anterior tibial translation with a soft endpoint. The pivot‐shift test was positive (grade 1+), indicating mild dynamic rotational instability. Posterior drawer and posterolateral drawer tests were negative. No varus or valgus laxity was observed at either 0° or 30° of knee flexion. The dial test was negative at both 30° and 90° of knee flexion. The McMurray test was negative.

The differential diagnosis included meniscal injury, medial or lateral collateral ligament sprain, and posterolateral corner injury; however, the absence of joint‐line tenderness, stable collateral ligaments, and negative provocative tests made these less likely, and the positive Lachman, anterior drawer, and pivot‐shift tests pointed strongly to anterior cruciate ligament disruption.

### 2.3 Diagnostic Assessment

**FIGURE 2:**
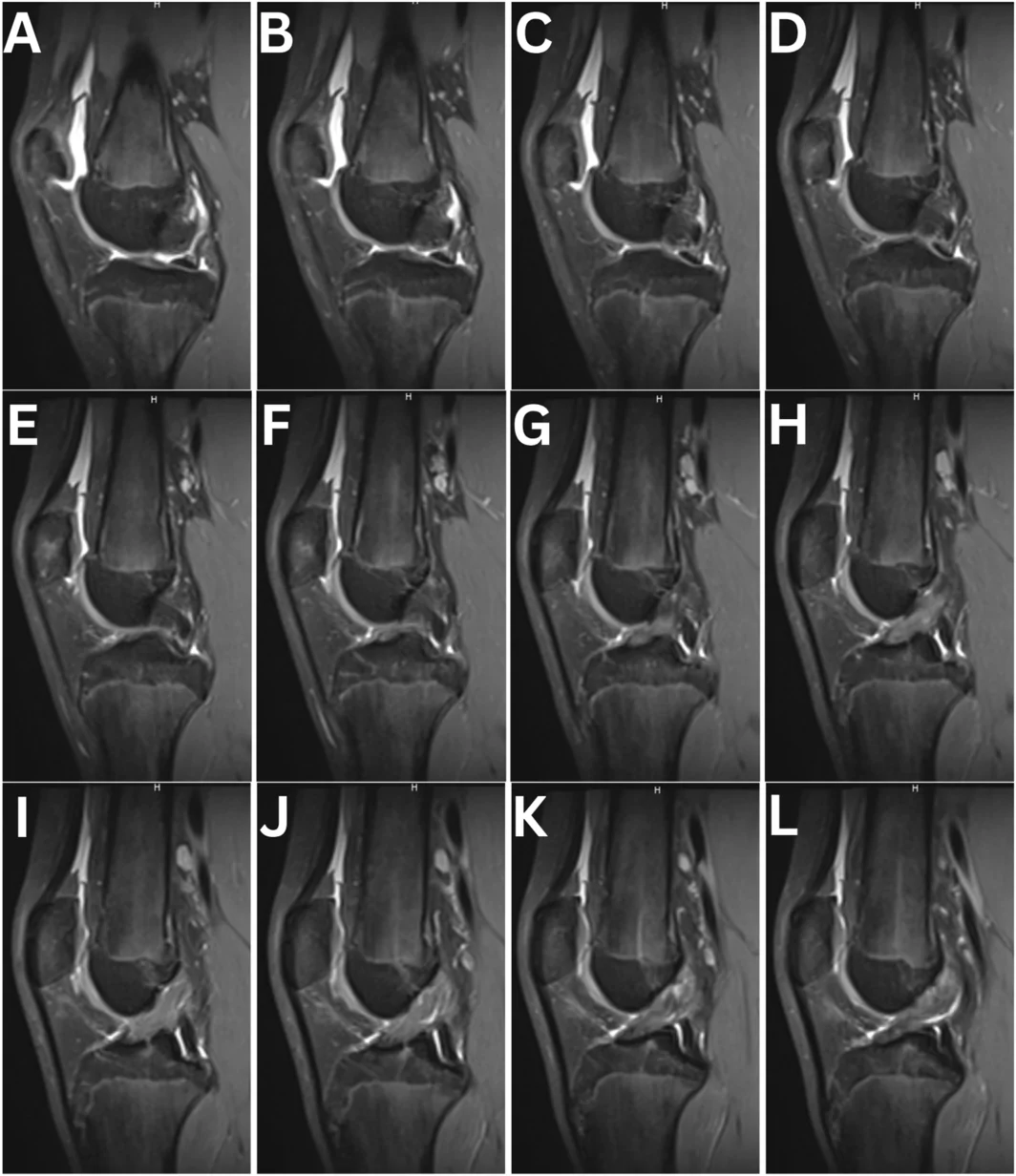
Initial sagittal MRI. Consecutive sagittal fat‐suppressed proton density–weighted images of the right knee (A–L, medial to lateral) show a high‐grade ACL tear with features of complete rupture, including loss of normal fiber continuity, fluid in the intercondylar notch, and a thickened, wavy, horizontalized remnant with increased intrasubstance signal. Joint effusion is present, while the PCL is intact. Overall, the findings are most consistent with complete ACL rupture, although a high‐grade partial tear cannot be excluded.

**FIGURE 3:**
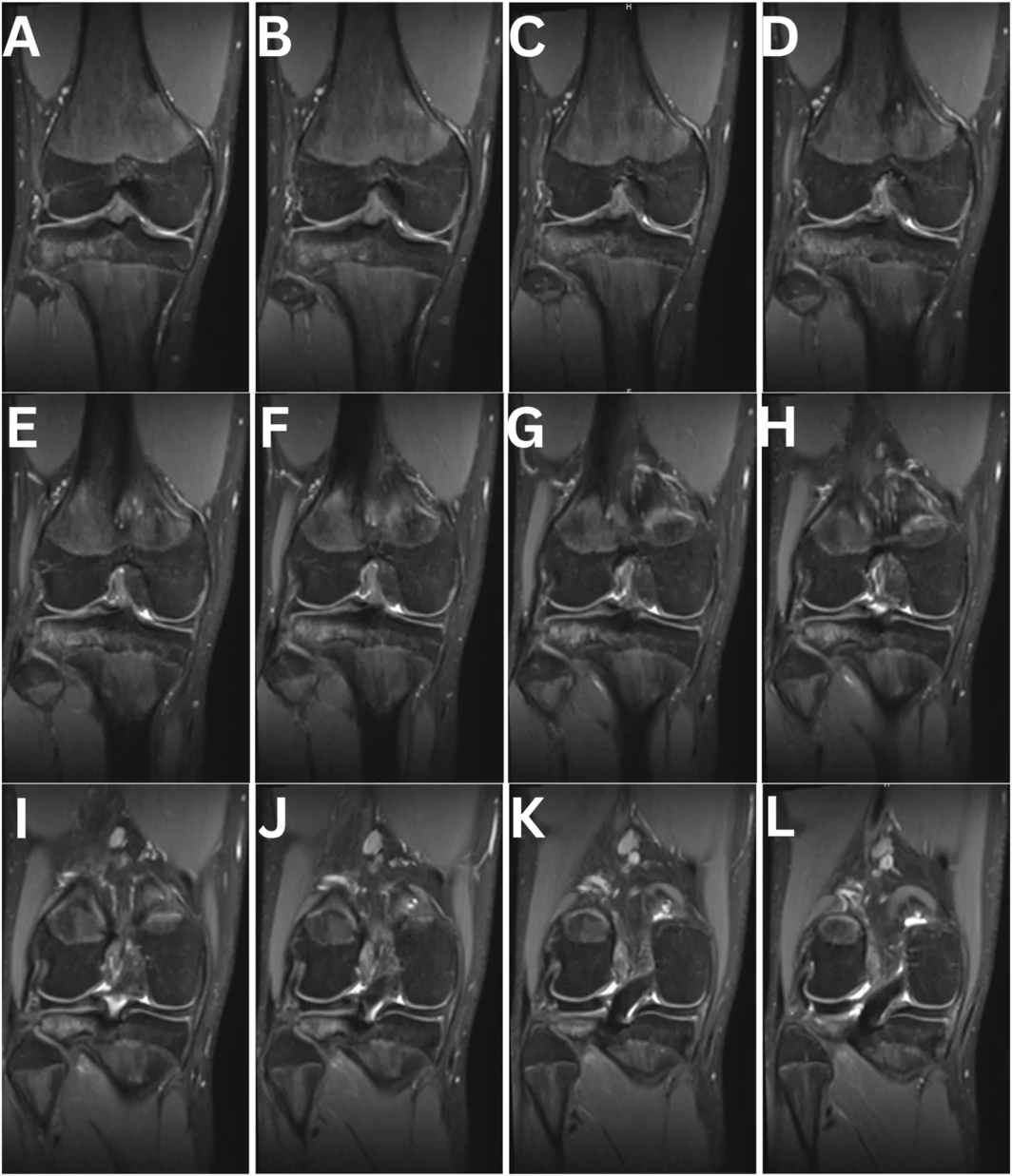
Initial coronal MRI. Coronal fat‐suppressed proton density–weighted images (A–L, anterior to posterior) show absence of the normal taut ACL band on mid‐notch slices (E–J), with heterogeneous tissue and fluid signal in the intercondylar notch consistent with a torn ACL remnant. Bone‐marrow edema is present in the lateral femoral condyle and lateral/posterolateral tibial plateau (A–H). Joint effusion is present. The PCL and collateral ligaments remain intact, with no displaced fracture or Segond‐type avulsion.

**FIGURE 4:**
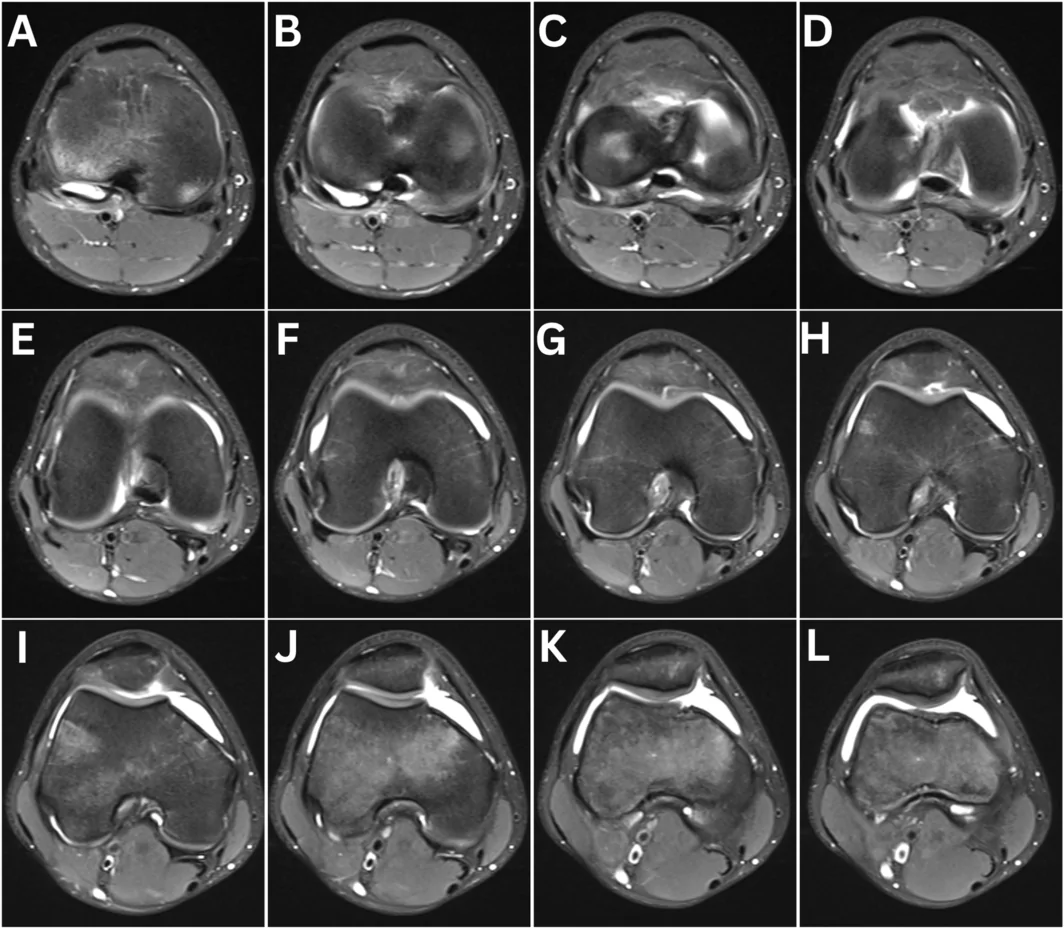
Initial axial MRI. Axial T2‐weighted fat‐suppressed images (A–L, proximal tibia to distal femur) show absence of the normal ACL band, with heterogeneous high signal in the intercondylar notch consistent with a torn, edematous ligament. Associated pivot‐shift bone bruising of the lateral femoral condyle and posterolateral tibial plateau, with joint effusion, is present.

Standard anteroposterior and lateral radiographs of the right knee obtained at presentation demonstrated normal alignment without evidence of fracture, osteochondral defect, or bony avulsion. Magnetic resonance imaging was performed within 2 weeks of injury using a 1.5‐T Philips scanner and included standard sagittal, coronal, and axial proton density–weighted and T2‐weighted sequences characterized by loss of normal fiber continuity, absence of discernible continuous fibers, and abnormal ligament discontinuity and orientation across multiple planes (Figures 2, 3, 4). No continuous ACL fibers were identified spanning the femoral and tibial attachments, with a retracted proximal ACL remnant evident. Mild joint effusion was also present. Using the MRI‐based method described by Harmes et al. for measuring static anterior tibial subluxation, static tibial translation measured 10.82 mm in the lateral compartment and 2.99 mm in the medial compartment [16] (Figure S1A,B).

**FIGURE 5:**
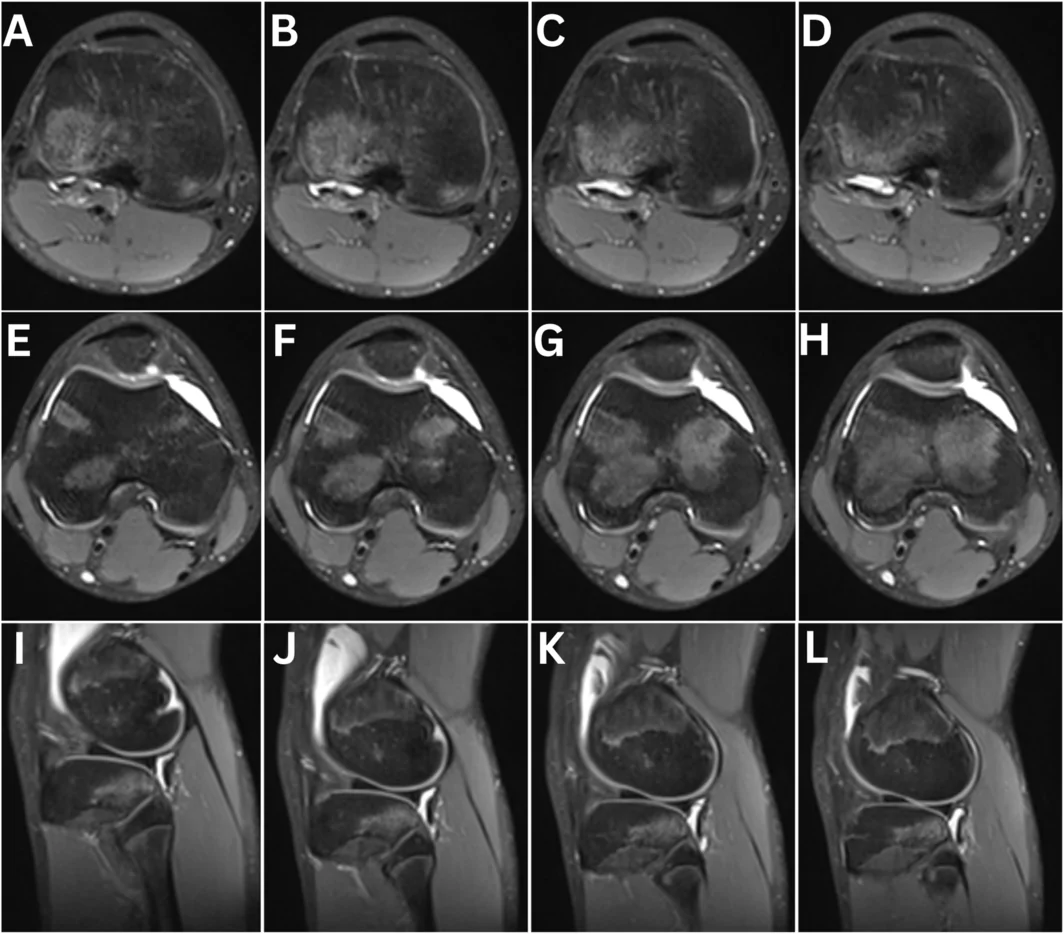
Initial axial MRI showing pivot‐shift bone bruising. Fat‐suppressed fluid‐sensitive images demonstrate patchy bone marrow edema in the posterolateral tibial plateau, extending into the proximal tibial metaphysis (A–D), and corresponding edema in the femoral condyles, particularly the lateral femoral condyle (E–H). Additional sagittal images (I–L) demonstrate the sagittal distribution of the lateral femoral condyle bone contusion.

On axial sequences, the femoral contusion was centered in the mid‐portion of the lateral femoral condyle, involving the terminal sulcus region and adjacent weight‐bearing surface, immediately superior to the anterior horn of the lateral meniscus (Figure S2), with associated edema in the posterolateral tibial plateau. On sagittal sequences, the lateral femoral condyle contusion was predominantly located within the weight‐bearing portion of the condyle, posterior to the level of the anterior horn of the lateral meniscus (Figure 5). The tibial ACL footprint cortex was intact, with no avulsion fragment and no fluid cleft tracking into the tibial insertion, arguing against a tibial‐sided avulsion‐type injury.

The popliteus tendon appeared intact, although there was a mild increase in signal at the myotendinous junction, suggestive of low‐grade strain. The quadriceps and patellar tendons were normal, and the medial and lateral retinacula were intact. Patellar cartilage and Hoffa's fat pad appeared normal, and surrounding muscles, vessels, and nerves were unremarkable. No associated meniscal or chondral injuries were identified.

During reassessment of the original MRI, certain coronal images demonstrated features suggestive of predominant posterolateral bundle involvement near the femoral attachment, with possible preservation of fibers corresponding to the anteromedial bundle. The relevant coronal image was compared with its corresponding sagittal image (Figure S3). Although the paired images suggested possible predominant posterolateral bundle involvement, the individual bundle morphology could not be determined confidently; therefore, no definitive bundle‐specific injury pattern was assigned.

### 2.4 Initial Management and Treatment Decision

Based on clinical examination and MRI findings, a diagnosis of a functionally complete ACL rupture was established at our center, with the indication for reconstruction supported by a formal musculoskeletal radiology report. Due to the patient's reluctance to undergo surgery, he deferred the procedure and sought multiple independent specialist opinions. Two knee‐specialized orthopedic surgeons independently reviewed the clinical and imaging data and concurred with both the diagnosis and the recommendation for ACL reconstruction. Given the patient's age, athletic demands, and imaging profile, a consensus plan was established to proceed with reconstruction following a structured rehabilitation program. The injury was specifically classified as a functionally complete rupture, reflecting the combination of clinical instability and MRI evidence rather than histologic confirmation of a full‐thickness tear.

### 2.5 Follow‐Up Assessment and Outcome

Ultimately, the patient elected against surgical intervention and did not attend scheduled follow‐up visits. He re‐presented approximately 1 year post‐injury. During the first 12 months, he did not participate in any structured rehabilitation but engaged in unstructured activity modification (avoiding pivoting and cutting), progressively reducing his sports participation and completely stopping athletic activities within the first month post‐injury. He denied persistent pain, swelling, or recurrent instability during activities of daily living and described progressive subjective improvement in knee function, noting complete confidence during non‐sporting activities. Physical examination revealed a clinically stable knee. The Lachman test was normal with a firm endpoint; the anterior drawer test was negative; the pivot‐shift test was negative; active and passive range of motion were full and symmetric compared with the contralateral knee, without pain at terminal flexion. No joint effusion was present. Quadriceps strength was symmetric. Instrumented laxity testing (e.g., KT‐1000, KT‐2000, or Rolimeter) was not available at our center at the time of evaluation. A single‐leg hop‐for‐distance test was also performed as part of the functional assessment to evaluate dynamic knee control and tolerance of single‐leg loading. However, the quantitative hop distance and corresponding limb symmetry index were not documented in the retrospective clinical records and therefore could not be reported. Qualitatively, the patient completed the task on the involved limb without pain, apprehension, or observable dynamic knee valgus during landing. Given the absence of quantitative hop‐test data, this assessment was interpreted as supportive clinical information rather than a formal objective measure of functional performance. Given this unexpected clinical stability, a repeat MRI was obtained to reassess ligament integrity before proceeding with the planned reconstruction.

**FIGURE 6:**
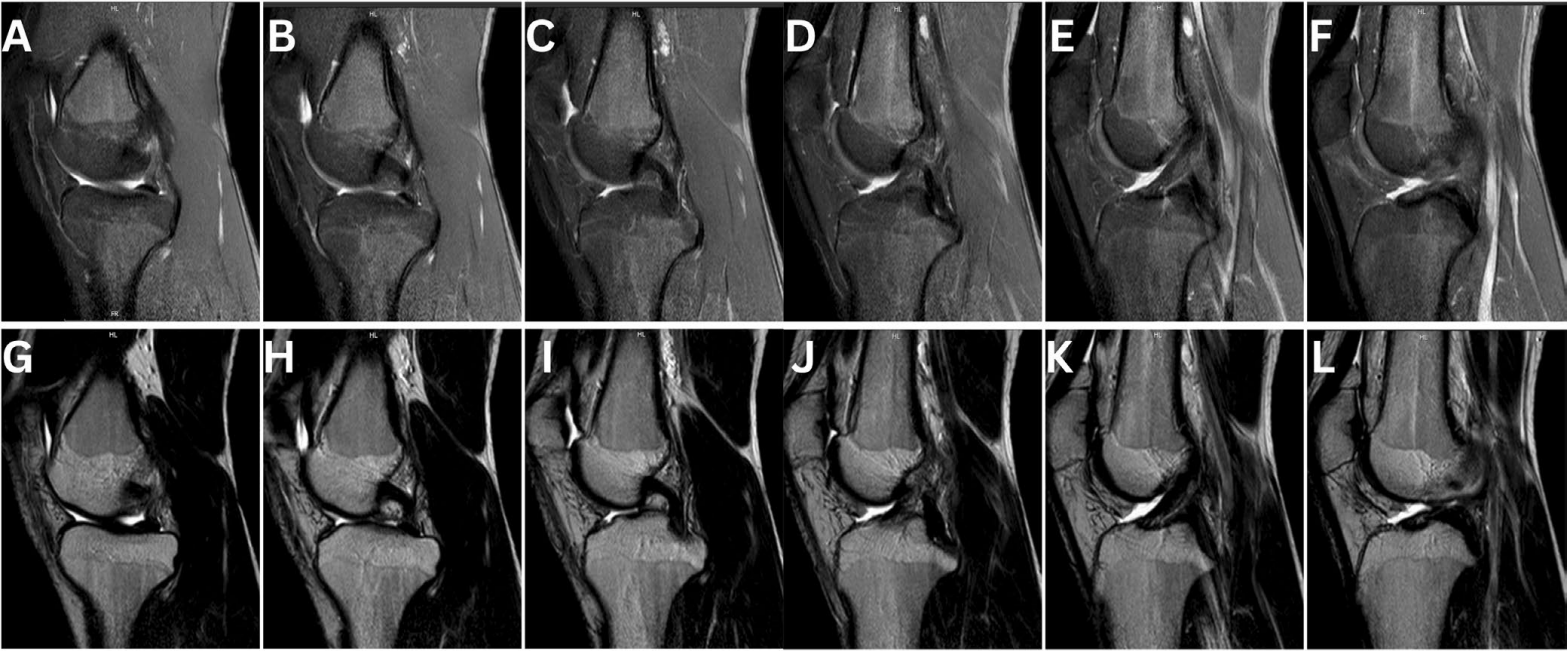
Follow‐up sagittal MRI at 12 months. Fat‐suppressed proton density–weighted (A–F) and non‐fat‐suppressed T2‐weighted (G–L) images show restoration of a continuous, taut, low‐signal ACL band with normal oblique orientation and intact femoral and tibial attachments. The prior discontinuity, fluid‐filled notch, and wavy ligament remnant are no longer evident. Only trace joint fluid is present; the PCL and menisci are unremarkable.

**FIGURE 7:**
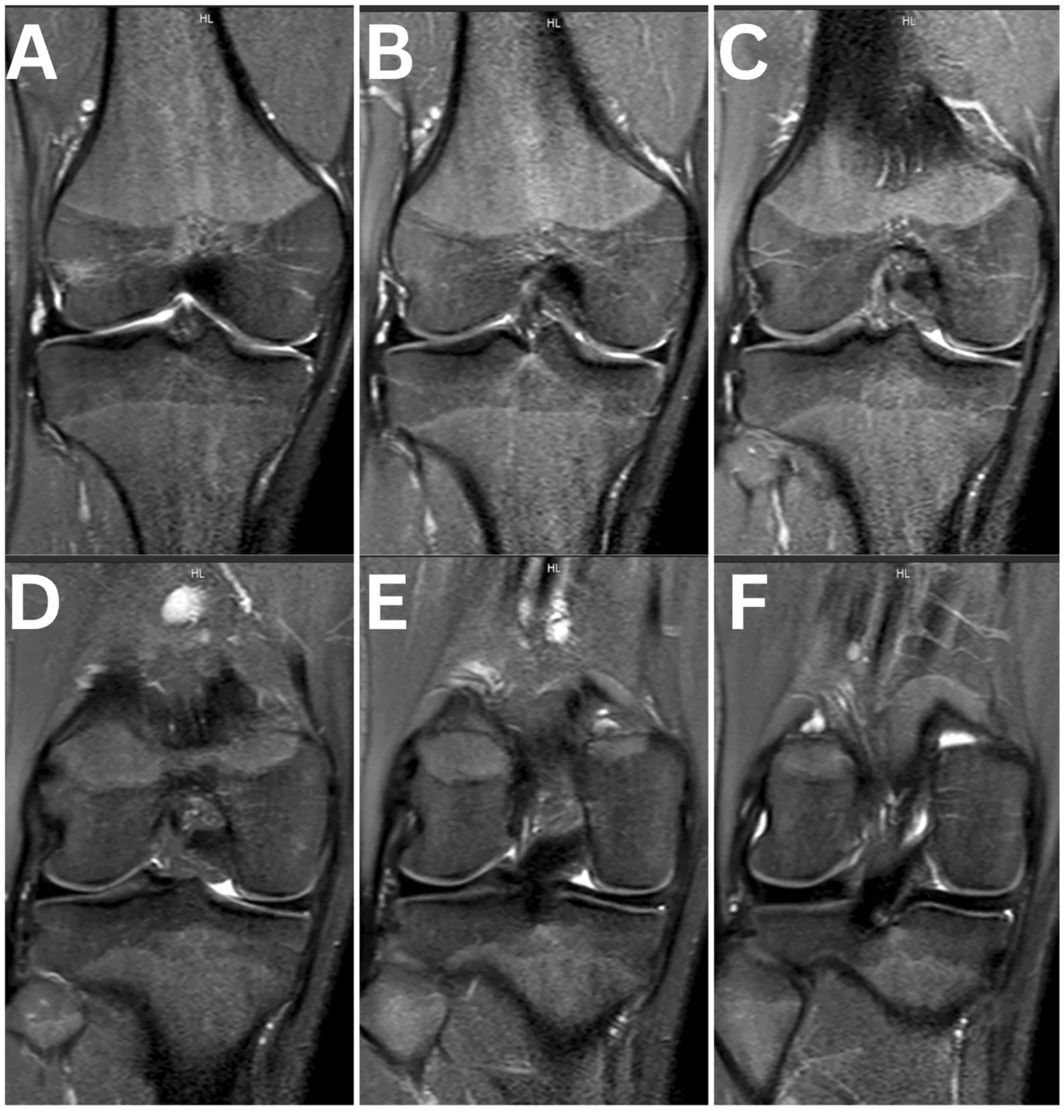
Follow‐up coronal MRI at 12 months. Coronal fat‐suppressed proton density–weighted images (A–F, anterior to posterior) demonstrate restoration of ACL continuity, with a continuous low‐signal band of normal oblique orientation extending from the femoral notch to the tibial intercondylar region. The prior empty‐notch appearance and heterogeneous ligament remnant are absent. Only trace joint fluid is present; the PCL, collateral ligaments, and menisci are unremarkable.

**FIGURE 8:**
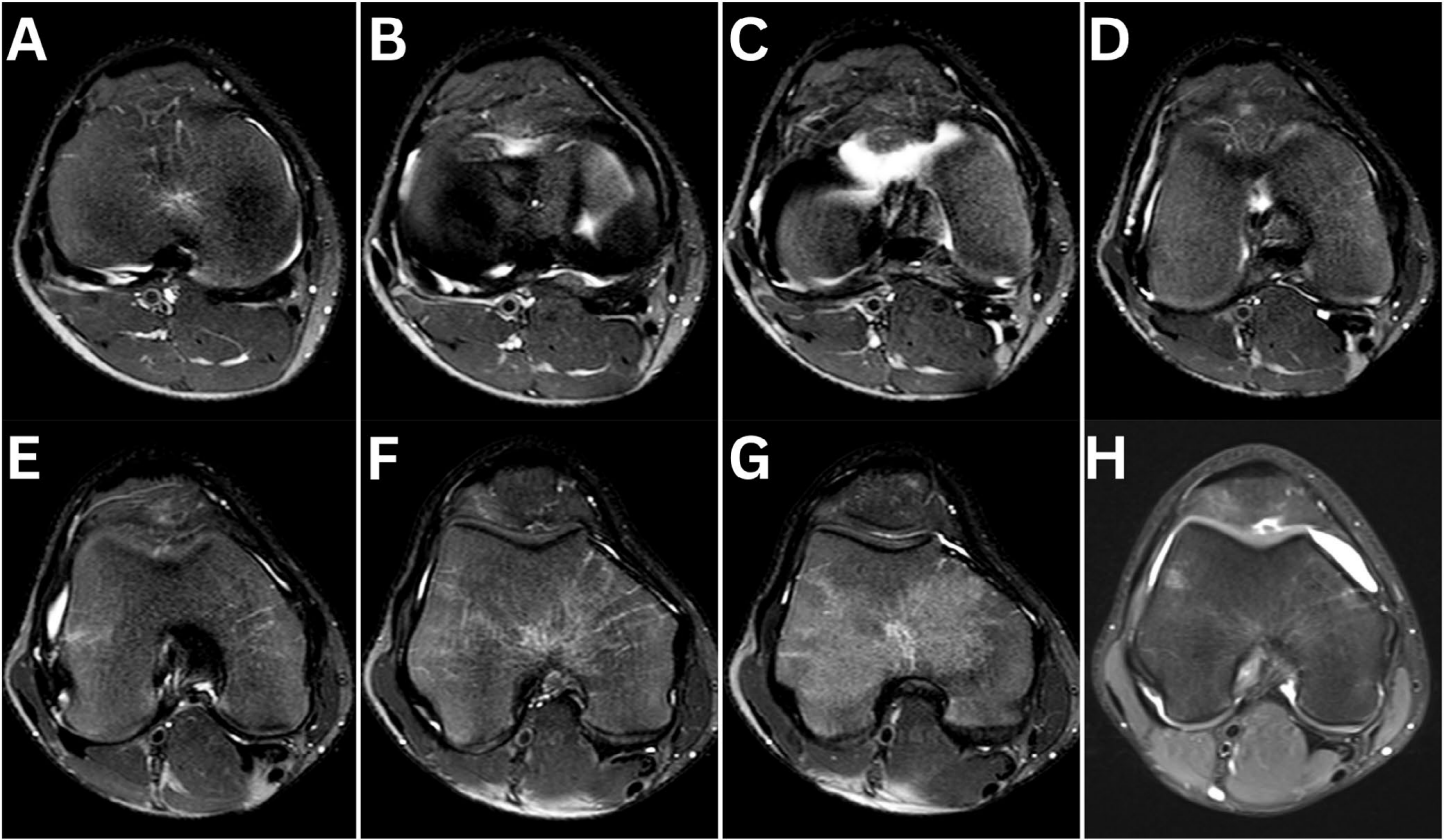
Follow‐up axial MRI at 12 months. T2‐weighted fat‐suppressed images (A–H) demonstrate restoration of ACL continuity, with a continuous, taut, low‐signal ligament band of normal oblique orientation within the intercondylar notch (D–G). The prior pivot‐shift bone‐marrow edema has resolved, with only trace physiologic joint fluid and no secondary signs of ACL deficiency.

The follow‐up MRI demonstrated continuous low‐signal ACL fibers with restored orientation, compatible with interval reconstitution of ligament continuity and absence of secondary signs of ACL deficiency (Figures 6, 7, 8). The morphology of the ACL on follow‐up MRI was further assessed using the measurement method described by Brockmeyer et al. [17], with ACL length measured from the most anterior to the posterosuperior aspect of the ligament and anteroposterior width measured orthogonally to the ACL at its midpoint. The restored ACL measured 40.18 mm in length and 4.37 mm in AP width (Figure S1C).

Given the normal clinical examination and radiologic findings, surgical intervention was deferred. A structured strength and conditioning program focusing on quadriceps and hamstring strengthening, neuromuscular control, and gradual return‐to‐sport preparation was initiated. The patient was enrolled in a supervised, progressive rehabilitation program emphasizing quadriceps and hamstring strengthening, neuromuscular control, proprioceptive training, and gradual functional loading of the knee. The program prioritized closed‐chain exercises, core stability, and movement pattern retraining while avoiding early, high‐risk pivoting and cutting maneuvers.

Throughout rehabilitation, the patient remained asymptomatic, reporting no episodes of knee pain, swelling, giving way, or subjective instability during activities of daily living or exercise progression. Serial examinations showed persistent knee stability with a firm Lachman endpoint, no pivot shift, symmetric anterior translation, full range of motion, and no joint effusion at follow‐up.

Manual muscle testing demonstrated normal quadriceps and hamstring strength (5/5) bilaterally, with no evidence of compensatory movement patterns. However, isokinetic dynamometry was not available to objectively quantify muscle strength or peak torque. The patient demonstrated good neuromuscular control during single‐leg squat and dynamic balance tasks, with no valgus collapse or apprehension. Progressive loading and low‐impact agility drills were tolerated without recurrence of symptoms.

At approximately 14 months after the initial injury and several months after initiation of structured rehabilitation, the patient had not yet returned to competitive soccer. This decision was made collaboratively based on a conservative return‐to‐sport strategy rather than ongoing symptoms or instability. He was preparing for a gradual, supervised return to pivoting sports, with emphasis on continued strength optimization, neuromuscular conditioning, and movement quality.

After the supervised rehabilitation program, approximately 15 months after the initial injury, the patient had successfully progressed through a graded return‐to‐sport protocol and returned to soccer training without pain, subjective instability, or recurrent episodes of giving way. He tolerated pivoting and cutting activities without symptom recurrence and did not require ACL reconstruction.

At a telephone follow‐up conducted approximately 4 years after the initial injury, the patient reported that he had continued soccer training for approximately 3 months following his documented return to training and then voluntarily discontinued the sport at 18 months post‐injury. This decision was attributed to fear of reinjury rather than persistent knee symptoms or instability. Thereafter, he engaged regularly in gym‐based resistance training and general fitness activities. No recurrent episodes of knee instability, knee swelling, additional knee injuries, or subsequent surgical intervention were reported. These findings suggest that the favorable clinical outcome observed during the in‐person follow‐up was maintained over the longer term, although return to the preinjury level of competitive participation was not achieved.

Written informed consent for publication of this case report and accompanying images was obtained from the patient's parent/legal guardian, and verbal assent was obtained from the patient.

## 3 Discussion

This case documents the unexpected clinical and radiologic resolution of functional ACL insufficiency in an adolescent athlete initially diagnosed with complete ACL rupture. While ACL reconstruction remains the standard of care for young athletes with instability [18, 19], accumulating evidence suggests that ligament healing may be biologically possible in selected cases [6, 7, 8, 9, 10, 11, 20, 21, 22]. The present report contributes to this evolving paradigm by demonstrating interval restoration of ACL continuity and stability following an initial presentation with clinical and imaging features typically associated with complete rupture.

Contemporary evidence indicates that ACL healing can occur through scar‐mediated ligament continuity, particularly in proximal tears with an intact synovial envelope. Favorable conditions include femoral‐sided rupture patterns, partial tears, stump preservation, reduced posterior tibial slope, and an environment that permits fibroblast migration and capillary ingrowth [6, 7, 8, 9, 10, 11, 21, 22, 23]. Experimental work further highlights the importance of joint kinematics; Kokubun et al. demonstrated that controlling abnormal tibial motion promotes spontaneous healing [24].

Importantly, this case lacked several of the favorable conditions outlined above, most notably early bracing or structured activity restriction. Similar observations have been reported in only a small number of skeletally immature patients. Bagsby et al. described intrinsic ACL healing in an adolescent managed nonoperatively, while Arts et al. reported MRI and clinical evidence of ACL regeneration in an 8‐year‐old child following conservative treatment with bracing [12, 13]. Notably, both reports involved younger patients and treatment strategies that included protection of the injured knee. In contrast, our patient was 16 years old and did not undergo early immobilization or structured bracing. These differences suggest that spontaneous restoration of ACL continuity may occur across a broader spectrum of adolescent patients and injury‐management pathways than previously recognized.

The relatively mild acute presentation, characterized by minimal effusion and preserved early function, combined with adolescent physiology, may have created a biological environment favorable for scar‐mediated healing. Adolescents potentially possess greater intrinsic healing capacity than adults [25, 26, 27], which could have offset the absence of bracing. However, human data on adolescent ACL healing are conflicting: MRI studies have shown poorer morphological healing in patients under 20 compared with adults [14], and adolescents may experience lower healing rates of associated anterolateral ligament injuries and greater residual rotational laxity after reconstruction [28].

The quantitative assessment of static tibial translation further supported the severity of the initial injury. Using the MRI‐based method described by Harmes et al., the initial lateral and medial compartment translations were 10.82 and 2.99 mm, respectively, compared with mean values of 6.8 and 2.5 mm, respectively, reported for adults with complete ACL injuries in their cohort [16]. Although these values are not normative and the reference population was adult, the greater lateral translation in our patient provides additional quantitative evidence of substantial anterior tibial subluxation associated with the initial complete ACL disruption.

The initial MRI also demonstrated an extended bone‐contusion pattern involving the femur and tibia, with features resembling bone‐bruising patterns described in association with ACL injury. In particular, the femoral component extended immediately superior to the anterior horn of the lateral meniscus, a location described in association with ACL injury [29, 30, 31]. However, the diagnostic significance of bone bruising should be interpreted cautiously. Zeiss et al. reported that bone contusions were more frequent in complete than partial ACL tears, but found no difference in their general appearance or location between the two groups [32]. Thus, in the present case, the overall bone‐contusion pattern provides supportive evidence of an ACL injury but cannot independently establish complete ACL rupture.

Beyond restoration of fiber continuity, the restored ACL measured 40.18 mm in length and 4.37 mm in AP width. The measured length was comparable to the normative MRI measurements reported by Brockmeyer et al. for male adolescents aged 16–18 years, who had a mean ACL length of 38.90 ± 4.47 mm [17]. However, the AP width of the restored ACL was smaller than the reported mean of 6.41 ± 0.56 mm [17]. Thus, although the restored ACL demonstrated continuous fiber architecture, appropriate orientation, and a longitudinal dimension comparable to that of native ACLs in an age‐ and sex‐matched population, its AP width remained below the reported normative range. These findings provide morphometric support for substantial restoration of ACL continuity, while indicating that the restored ligament was not completely normalized in all dimensions.

An important alternative explanation is that the initial injury represented a high‐grade partial tear rather than a complete rupture. This possibility cannot be entirely excluded because arthroscopic confirmation was not performed. Nevertheless, the initial MRI demonstrated nonvisualization of the continuous ACL fibers on sagittal sequences and an apparent empty‐notch appearance within the intercondylar region, findings interpreted as consistent with complete ACL disruption. These imaging findings, together with concordant clinical instability, supported a diagnosis of functional ACL insufficiency at presentation. Independent review by multiple experienced clinicians further reduced the likelihood of diagnostic misclassification.

While MRI findings were consistent with a complete ACL rupture, the inability of conventional MRI to distinguish high‐grade partial tears from complete disruptions reliably must be acknowledged. Van Dyck et al. showed that partial ACL tears frequently demonstrate imaging characteristics overlapping with complete ruptures, mucoid degeneration, or even normal‐appearing ligaments, underscoring the limitations of MRI as a definitive diagnostic reference in isolation [33]. Accordingly, this report describes a case of functional ACL insufficiency with subsequent clinical and radiologic recovery, rather than definitive histologic regeneration of the ligament. Nevertheless, caution is warranted. Spontaneous ACL healing is not predictable, and long‐term outcomes, including the risk of reinjury and degenerative changes, remain uncertain. Careful patient selection, close follow‐up, and shared decision‐making are essential.

Although the patient returned to soccer training without recurrent instability, he did so approximately 15 months after the initial injury and ultimately did not resume semi‐professional competitive soccer. Importantly, this decision was attributed to fear of reinjury rather than persistent pain, instability, or objective functional limitations. After several months of soccer training, he voluntarily transitioned to other forms of physical activity, including gym‐based resistance training and general fitness exercise. Many adolescents undergoing ACL reconstruction with contemporary, criterion‐based rehabilitation may return to knee‐strenuous or competitive sport around 9–12 months [34]. Therefore, the present case should not be interpreted as demonstrating a faster or equivalent return‐to‐sport outcome compared with surgical management. Rather, it illustrates that durable restoration of knee stability and function may be achieved without ACL reconstruction in selected patients. However, the recovery trajectory may be prolonged, and a return to the preinjury level of competitive sport may remain influenced by psychological readiness as well as physical recovery.

In the present case, the patient's decision to discontinue competitive soccer was explicitly attributed to fear of reinjury despite restoration of ACL continuity, clinical stability, and absence of persistent symptoms or functional limitations. Fear of reinjury is a well‐recognized barrier to return to sport after ACL injury. A systematic review and meta‐analysis found that fear of reinjury was the most frequently cited reason for failure to return to sport [35]. This concern is also prominent among adolescents, with one cohort reporting that 27% did not return to or sustain participation in their primary sport, with fear of another injury among the reported reasons [36]. Thus, the present case further illustrates that restoration of anatomical continuity and clinical stability may not be sufficient to overcome psychological barriers to return to sport.

## 4 Limitations

This report has several limitations. Arthroscopic confirmation of ligament integrity was not performed, and MRI cannot definitively differentiate between scar‐mediated healing and true anatomic regeneration. In addition, objective instrumented assessment of anterior knee laxity, such as KT‐1000, KT‐2000, or Rolimeter testing, was not performed at either the initial or follow‐up evaluations. Consequently, restoration of knee stability was determined using standard clinical examination findings, including Lachman and pivot‐shift testing, rather than quantitative measurements of anterior tibial translation. Although the follow‐up examination demonstrated no detectable clinical instability and was concordant with MRI evidence of restored ACL continuity, the absence of instrumented laxity measurements limits the ability to objectively quantify the degree of biomechanical recovery. Similarly, isokinetic dynamometry was not available to objectively quantify muscle performance or peak torque. The absence of validated patient‐reported outcome measures, such as the International Knee Documentation Committee (IKDC) score, further limits the comprehensive characterization of functional recovery. Additionally, long‐term outcomes following return to high‐level pivoting sports remain unknown. These limitations underscore the need for cautious interpretation and highlight the importance of prospective cohort studies incorporating standardized imaging, instrumented knee laxity assessment, isokinetic strength testing, validated patient‐reported outcome measures, and long‐term follow‐up.

## 5 Conclusion

This case demonstrates that restoration of clinical stability and radiologic ACL continuity can occur in selected adolescent athletes who initially present with functional ACL rupture. However, this outcome should not be interpreted as demonstrating equivalence to surgical management, as the patient did not return to competitive sport within the typical postoperative timeframe. While surgical reconstruction remains the standard treatment for young athletes with persistent instability, this report emphasizes the importance of individualized decision‐making and timely reassessment before definitive intervention. Prospective studies are needed to identify clinical, biomechanical, and imaging predictors of successful nonoperative outcomes and to define the long‐term implications of spontaneous ACL healing in high‐demand populations.

## Author Contributions

**Hooman Angoorani:** conceptualization, methodology, data curation, supervision, writing – review and editing, investigation. **Molood Jafari Fesharaki:** conceptualization, investigation, methodology, project administration, writing – review and editing, data curation. **Chakavak Valadkhani:** visualization, writing – review and editing, investigation, methodology, software, writing – original draft. **Hossein Safaei:** investigation, writing – original draft, writing – review and editing, visualization, validation, software, methodology.

## Funding

The authors have nothing to report.

## Disclosure

No copyrighted material from other sources requiring permission for reproduction has been used in this manuscript. This manuscript is a case report and does not describe a clinical trial.

## Ethics Statement

Ethical approval was not required for this retrospective case report in accordance with institutional policy. All procedures performed were conducted in accordance with the ethical standards of the institutional and national research committee and with the principles of the Declaration of Helsinki and its subsequent amendments.

## Consent

Written informed consent for publication of this case report and accompanying images was obtained from the patient's parent/legal guardian, as the patient was a minor at the time consent was provided. The patient also provided verbal assent.

## Conflicts of Interest

The authors declare no conflicts of interest.

## Supporting information


**Figure S1:** MRI‐based quantitative assessment of tibial translation and restored ACL morphology. (A, B) Initial MRI demonstrating measurement of static anterior tibial translation according to the method described by Harmes et al. Lateral compartment translation measured 10.82 mm, while medial compartment translation measured 2.99 mm. (C) Follow‐up MRI demonstrating measurement of the restored ACL according to the method described by Brockmeyer et al., with ACL length measured from the most anterior to the posterosuperior aspect of the ligament and anteroposterior width measured orthogonally at the midpoint. The restored ACL measured 40.18 mm in length and 4.37 mm in anteroposterior width.
**Figure S2:** Lateral femoral condyle bone bruise on initial MRI. Sagittal fat‐suppressed proton density–weighted image demonstrates bone bruising involving the lateral femoral condyle, including a component immediately superior to the anterior horn of the lateral meniscus (red line).
**Figure S3:** Coronal and sagittal MRI assessment of the ACL femoral attachment and bundle morphology. Coronal and corresponding sagittal images from the initial MRI suggested possible posterolateral bundle involvement near the femoral attachment, with possible preservation of some anteromedial fibers. However, the available sequences did not permit confident differentiation of individual ACL bundles. The images are presented to document the cross‐sectional assessment rather than establish a definitive bundle‐specific injury pattern.

## Data Availability

The data supporting the findings of this case report are available from the corresponding author upon reasonable request. To protect patient privacy, individual‐level data will not be publicly shared.

## References

[1] L. Siegel , C. Vandenakker‐Albanese , and D. Siegel , “Anterior Cruciate Ligament Injuries: Anatomy, Physiology, Biomechanics, and Management,” Clinical Journal of Sport Medicine 22, no. 4 (2012): 349–355, 10.1097/JSM.0b013e3182580cd0. 22695402

[2] N. A. Friel and C. R. Chu , “The Role of ACL Injury in the Development of Posttraumatic Knee Osteoarthritis,” Clinics in Sports Medicine 32, no. 1 (2013): 1–12, 10.1016/j.csm.2012.08.017. 23177457 PMC6548436

[3] E. R. Laskowski , “ACL Injury and Rehabilitation,” Current Physical Medicine and Rehabilitation Reports 2 (2014): 35–40, 10.1007/s40141-013-0036-8.

[4] R. H. Brophy and K. J. Lowry , “American Academy of Orthopaedic Surgeons Clinical Practice Guideline Summary: Management of Anterior Cruciate Ligament Injuries,” Journal of the American Academy of Orthopaedic Surgeons 31, no. 11 (2023): 531–537, 10.5435/JAAOS-D-22-01020. 36727995 PMC10168113

[5] J. Liang , Y. Luo , Y. Yang , et al., “Global Overview of Anterior Cruciate Ligament Reconstruction in Children and Adolescents Over the Past 20 Years: A Bibliometric Analysis,” Journal of Orthopaedic Surgery and Research 19 (2024): 350, 10.1186/s13018-024-04829-2. 38867234 PMC11170893

[6] S. R. Filbay , F. W. Roemer , L. S. Lohmander , et al., “Evidence of ACL Healing on MRI Following ACL Rupture Treated With Rehabilitation Alone May Be Associated With Better Patient‐Reported Outcomes: A Secondary Analysis From the KANON Trial,” British Journal of Sports Medicine 57, no. 2 (2023): 91–98, 10.1136/bjsports-2022-105473. 36328403 PMC9872245

[7] F. Blanke , K. Trinnes , N. Oehler , et al., “Spontaneous Healing of Acute ACL Ruptures: Rate, Prognostic Factors and Short‐Term Outcome,” Archives of Orthopaedic and Trauma Surgery 143, no. 7 (2023): 4291–4298, 10.1007/s00402-022-04701-0. 36515708 PMC10293391

[8] D. R. Baka , E. Thuo , V. Barshay , and D. S. Yun , “A Rare Case of Spontaneous Healing of an Anterior Cruciate Ligament Tear,” Cureus 17, no. 3 (2025): e80171, 10.7759/cureus.80171. 40190967 PMC11972121

[9] L. Previ , E. Monaco , A. Carrozzo , et al., “Spontaneous Healing of a Ruptured Anterior Cruciate Ligament: A Case Series and Literature Review,” Journal of Experimental Orthopaedics 10, no. 1 (2023): 11, 10.1186/s40634-022-00566-9. 36738386 PMC9898698

[10] G. A. Malanga , J. Giradi , and S. F. Nadler , “The Spontaneous Healing of a Torn Anterior Cruciate Ligament,” Clinical Journal of Sport Medicine 11, no. 2 (2001): 118–120, 10.1097/00042752-200104000-00010. 11403112

[11] M. Costa‐Paz , M. A. Ayerza , I. Tanoira , J. Astoul , and D. L. Muscolo , “Spontaneous Healing in Complete ACL Ruptures: A Clinical and MRI Study,” Clinical Orthopaedics and Related Research 470, no. 4 (2012): 979–985, 10.1007/s11999-011-1933-8. 21643922 PMC3293953

[12] S. J. Arts , M. P. J. Polak , and R. P. A. Janssen , “Anterior Cruciate Ligament Regeneration in an 8‐Year‐Old Patient,” Journal of Knee Surgery Reports 1, no. 1 (2015): 35–38, 10.1055/s-0034-1399757.

[13] D. Bagsby , G. Gantsoudes , and R. Klitzman , “Intrinsic Healing of the Anterior Cruciate Ligament in an Adolescent,” American Journal of Orthopedics 44, no. 8 (2015): E294–E297. 26251948

[14] H. Ihara and T. Kawano , “Influence of Age on Healing Capacity of Acute Tears of the Anterior Cruciate Ligament Based on Magnetic Resonance Imaging Assessment,” Journal of Computer Assisted Tomography 41, no. 2 (2017): 206–211, 10.1097/RCT.0000000000000515. 28045756 PMC5359784

[15] A. K. Ramachandran , J. S. Pedley , S. Moeskops , J. L. Oliver , G. D. Myer , and R. S. Lloyd , “Changes in Lower Limb Biomechanics Across Various Stages of Maturation and Implications for ACL Injury Risk in Female Athletes: A Systematic Review,” Sports Medicine 54, no. 7 (2024): 1851–1876, 10.1007/s40279-024-02022-3. 38671176 PMC11257789

[16] J. C. Harmes , S. T. Ubl , E. Koch , B. Bouillon , D. Günther , and T. R. Pfeiffer , “Complete ACL Injuries Are Associated With Higher Static Tibial Subluxation Than Partial ACL Injuries or Intact ACLs Measured on MRI,” Journal of Experimental Orthopaedics 12, no. 4 (2025): e70526, 10.1002/jeo2.70526. 41200436 PMC12587486

[17] M. Brockmeyer , S. Norrick , G. Wagenpfeil , J. Stroeder , and S. Landgraeber , “Size and Morphology of the Anterior and Posterior Cruciate Ligaments at Different Pediatric Age Intervals: An MRI Analysis,” Orthopaedic Journal of Sports Medicine 11, no. 10 (2023): 23259671231201642, 10.1177/23259671231201642. 37900865 PMC10612459

[18] H. Jin , N. Tahir , S. Jiang , et al., “Management of Anterior Cruciate Ligament Injuries in Children and Adolescents: A Systematic Review,” Sports Medicine ‐ Open 11, no. 1 (2025): 40, 10.1186/s40798-025-00844-7. 40263204 PMC12014893

[19] J. R. McCarroll , K. D. Shelbourne , and D. V. Patel , “Anterior Cruciate Ligament Injuries in Young Athletes. Recommendations for Treatment and Rehabilitation,” Sports Medicine 20, no. 2 (1995): 117–127, 10.2165/00007256-199520020-00006. 7481281

[20] B. Sas , A. Serner , P. D'Hooghe , and J. Arnaiz , “Extrasynovial ACL Tear as Potential Indicator for ACL Healing Capability: A Case Report of a Recreational Football Player With 3 Years of Serial Follow‐Up Tracking ACL Healing,” JOSPT Cases 3, no. 4 (2023): 212–218, 10.2519/josptcases.2023.11747.

[21] A. Pitsillides , D. Stasinopoulos , and K. Giannakou , “Healing Potential of the Anterior Cruciate Ligament in Terms of Fiber Continuity After a Complete Rupture: A Systematic Review,” Journal of Bodywork and Movement Therapies 28 (2021): 246–254, 10.1016/j.jbmt.2021.06.003. 34776148

[22] J. Roe , L. Salmon , A. Waller , J. Linklater , and L. Pinczewski , “Spontaneous Healing of the Ruptured Anterior Cruciate Ligament: A Case Series of 21 Patients,” Orthopaedic Journal of Sports Medicine 4, no. 7 (2016): 2325967116S00080, 10.1177/2325967116S00080.

[23] M. Kurosaka , S. Yoshiya , T. Mizuno , and K. Mizuno , “Spontaneous Healing of a Tear of the Anterior Cruciate Ligament. A Report of Two Cases,” Journal of Bone and Joint Surgery. American Volume 80, no. 8 (1998): 1200–1203, 10.2106/00004623-199808000-00015. 9730131

[24] T. Kokubun , N. Kanemura , K. Murata , et al., “Effect of Changing the Joint Kinematics of Knees With a Ruptured Anterior Cruciate Ligament on the Molecular Biological Responses and Spontaneous Healing in a Rat Model,” American Journal of Sports Medicine 44, no. 11 (2016): 2900–2910, 10.1177/0363546516654687. 27507845

[25] J. Nyland , M. N. Sirignano , J. Richards , and R. J. Krupp , “Regenerative Anterior Cruciate Ligament Healing in Youth and Adolescent Athletes: The Emerging Age of Recovery Science,” Journal of Functional Morphology and Kinesiology 9, no. 2 (2024): 80, 10.3390/jfmk9020080. 38804446 PMC11130880

[26] M. M. Murray , E. M. Magarian , S. L. Harrison , A. N. Mastrangelo , D. Zurakowski , and B. C. Fleming , “The Effect of Skeletal Maturity on Functional Healing of the Anterior Cruciate Ligament,” Journal of Bone and Joint Surgery. American Volume 92, no. 11 (2010): 2039–2049, 10.2106/JBJS.I.01368. 20810854 PMC2924734

[27] A. N. Mastrangelo , E. M. Magarian , M. P. Palmer , P. Vavken , and M. M. Murray , “The Effect of Skeletal Maturity on the Regenerative Function of Intrinsic ACL Cells,” Journal of Orthopaedic Research 28, no. 5 (2010): 644–651, 10.1002/jor.21018. 19890988 PMC2845722

[28] D. W. Lee , J. K. Lee , S. H. Kwon , et al., “Adolescents Show a Lower Healing Rate of Anterolateral Ligament Injury and a Higher Rotational Laxity Than Adults After Anterior Cruciate Ligament Reconstruction,” Knee 30 (2021): 113–124, 10.1016/j.knee.2021.03.020. 33894653

[29] J. P. Deangelis and K. P. Spindler , “Traumatic Bone Bruises in the Athlete's Knee,” Sports Health 2, no. 5 (2010): 398–402, 10.1177/1941738110377745. 23015967 PMC3445054

[30] V. Mandalia , A. J. Fogg , R. Chari , J. Murray , A. Beale , and J. H. Henson , “Bone Bruising of the Knee,” Clinical Radiology 60, no. 6 (2005): 627–636, 10.1016/j.crad.2005.01.014. 16038689

[31] S. Sohn , S. M. AlShammari , B. J. Hwang , and M. S. Kim , “A Systematic Review of Bone Bruise Patterns Following Acute Anterior Cruciate Ligament Tears: Insights Into the Mechanism of Injury,” Bioengineering 11, no. 4 (2024): 396, 10.3390/bioengineering11040396. 38671817 PMC11048204

[32] J. Zeiss , K. Paley , K. Murray , and S. R. Saddemi , “Comparison of Bone Contusion Seen by MRI in Partial and Complete Tears of the Anterior Cruciate Ligament,” Journal of Computer Assisted Tomography 19, no. 5 (1995): 773–776, 10.1097/00004728-199509000-00014. 7560324

[33] P. Van Dyck , E. De Smet , J. Veryser , et al., “Partial Tear of the Anterior Cruciate Ligament of the Knee: Injury Patterns on MR Imaging,” Knee Surgery, Sports Traumatology, Arthroscopy 20, no. 2 (2012): 256–261, 10.1007/s00167-011-1617-7. 21773827

[34] B. Thorolfsson , R. Piussi , T. Snaebjornsson , et al., “Greater Self‐Efficacy, Psychological Readiness and Return to Sport Amongst Paediatric Patients Compared With Adolescents and Young Adults, 8 and 12 Months After ACL Reconstruction,” Knee Surgery, Sports Traumatology, Arthroscopy 31, no. 12 (2023): 5629–5640, 10.1007/s00167-023-07623-5. PMC10719146 37861790

[35] K. Yensen , C. K. Mayfield , I. K. Bolia , et al., “Subjective Causes for Failure to Return to Sport After Anterior Cruciate Ligament Reconstruction: A Systematic Review and Meta‐Analysis,” Sports Health 17, no. 2 (2025): 243–251, 10.1177/19417381241231631. 38532528 PMC11569586

[36] L. Fones , R. O. Kostyun , A. D. Cohen , and J. L. Pace , “Patient‐Reported Outcomes, Return‐To‐Sport Status, and Reinjury Rates After Anterior Cruciate Ligament Reconstruction in Adolescent Athletes: Minimum 2‐Year Follow‐Up,” Orthopaedic Journal of Sports Medicine 8, no. 11 (2020): 2325967120964471, 10.1177/2325967120964471. 33283005 PMC7686622

