# Supplementary material for: Clinical and MRI Restoration of ACL Continuity Following Functional Complete ACL Rupture in an Adolescent Athlete: A Case Report
Source: Clin Case Rep. 2026 Sep 3;14(9):e73465. doi: 10.1002/ccr3.73465 (PMC13542709; doi:10.1002/ccr3.73465)
Supplement: Supplementary file 1 [file CCR3-14-e73465-s001.docx]

**Supplementary Figure S1. MRI-based quantitative assessment of tibial translation and restored ACL morphology.**
(A, B) Initial MRI demonstrating measurement of static anterior tibial translation according to the method described by Harmes et al. Lateral compartment translation measured 10.82 mm, while medial compartment translation measured 2.99 mm. (C) Follow-up MRI demonstrating measurement of the restored ACL according to the method described by Brockmeyer et al., with ACL length measured from the most anterior to the posterosuperior aspect of the ligament and anteroposterior width measured orthogonally at the midpoint. The restored ACL measured 40.18 mm in length and 4.37 mm in anteroposterior width.

**Supplementary Figure S2. Lateral femoral condyle bone bruise on initial MRI.** Sagittal fat-suppressed proton density–weighted image demonstrates bone bruising involving the lateral femoral condyle, including a component immediately superior to the anterior horn of the lateral meniscus (Red Line).

**Supplementary Figure S3. Coronal and sagittal MRI assessment of the ACL femoral attachment and bundle morphology.** Coronal and corresponding sagittal images from the initial MRI suggested possible posterolateral bundle involvement near the femoral attachment, with possible preservation of some anteromedial fibers. However, the available sequences did not permit confident differentiation of individual ACL bundles. The images are presented to document the cross-sectional assessment rather than establish a definitive bundle-specific injury pattern.
